# Anti-Aging Potential of a Novel Ingredient Derived from Sugarcane Straw Extract (SSE)

**DOI:** 10.3390/ijms25010021

**Published:** 2023-12-19

**Authors:** Maria João Carvalho, Sílvia Santos Pedrosa, Adélia Mendes, João Azevedo-Silva, João Fernandes, Manuela Pintado, Ana L. S. Oliveira, Ana Raquel Madureira

**Affiliations:** 1 CBQF—Centro de Biotecnologia e Química Fina—Laboratório Associado, Escola Superior de Biotecnologia, Universidade Católica Portuguesa, Rua Diogo Botelho 1327, 4169-005 Porto, Portugal; mjcarvalho@ucp.pt (M.J.C.); sspedrosa@ucp.pt (S.S.P.); marmendes@ucp.pt (A.M.); joao.pedro.silva@amyris.com (J.A.-S.); jfernandes@amyris.com (J.F.); mpintado@ucp.pt (M.P.); 2 Amyris Bio Products Portugal, Unipessoal Lda., Rua Diogo Botelho 1327, 4169-005 Porto, Portugal

**Keywords:** anti-aging, anti-pollution, cosmetics, hyaluronic acid, permeabilization

## Abstract

Natural and sustainable anti-aging ingredients have gained attention from the cosmetic industry. This study evaluated the anti-aging potential of a sugarcane straw extract-based (SSE) cosmetic ingredient. First, cytotoxicity tests were assessed in keratinocytes and fibroblast cell lines, and sensitization was carried out through the direct peptide reactivity assay. Subsequently, various anti-aging properties were investigated, including inhibiting skin aging-related enzymes, promoting elastin and hyaluronic acid synthesis, and anti-pollution activity. Finally, a permeability assay using a synthetic membrane resembling skin was conducted. The results demonstrated that the SSE ingredient effectively inhibited elastase (55%), collagenase (25%), and tyrosinase (47%) while promoting hyaluronic acid production at non-cytotoxic and low-sensitizer concentrations. Moreover, it reduced the inflammatory response provoked by urban pollution, as evidenced by decreased levels of IL1-α and IL-6. However, it was observed that the phenolic compounds predominantly reached the skin’s surface, indicating a limited ability to penetrate deeper layers of the skin. Therefore, it can be concluded that the SSE ingredient holds anti-aging properties, albeit with limited penetration into deeper skin layers. Further research and formulation advancements are needed to optimize the ingredient’s ability to reach and exert its effects in deeper skin layers.

## 1. Introduction

Skin aging is a prominent concern for modern consumers, influenced by intrinsic and extrinsic factors. Intrinsic aging is a natural process that occurs over time, while extrinsic aging is accelerated by environmental factors like UV radiation and smoking [1,2,3]. Exposure to these factors increases ROS, which provokes oxidative stress, causing damage to biological structures and leading to loss of cellular function, deep wrinkles, rough texture, and irregular pigmentation [4]. Skin structures, such as collagen, elastin, and hyaluronic acid, are affected by oxidative stress, which contributes to visible aging signs of the skin [5,6].

The cosmetic industry has witnessed a significant increase in interest in natural compounds. This shift is driven by concerns over the adverse reactions caused by synthetic ingredients, such as allergic contact dermatitis, irritant dermatitis, phototoxicity, and photoallergic reactions [1]. Moreover, natural cosmetic ingredients are known to be hypoallergenic and are absorbed more quickly by the skin. While using natural compounds is desirable, it is essential to ensure the sustainability of these resources. The preservation of natural resources should not be compromised by using natural ingredients. Therefore, a promising approach is to utilize industrial byproducts as sources of natural compounds, which can be considered an environmentally friendly alternative [7].

One compelling example of this practice is the utilization of sugarcane byproducts. Sugarcane is a widely cultivated crop, and its byproducts can be repurposed to extract valuable natural compounds for cosmetic applications. This approach reduces waste efficiently and aligns with sustainable practices [7]. By adopting this environmentally friendly strategy of using industrial byproducts as sources of natural compounds, the cosmetic industry can enhance the quality of its products while minimizing its ecological footprint. This approach showcases a commitment to consumer well-being and environmental sustainability, making it a positive trend in the industry. Sugarcane straw extract is a rich source of beneficial phenolic compounds, such as hydroxybenzoic acids, hydroxycinnamic, flavones, and organic acids [8]. These compounds contribute to the extract’s antioxidant and antimicrobial properties, making it a valuable ingredient for cosmetic applications [9,10]. Natural extracts containing phenolic compounds have been widely recognized for their potential anti-aging properties. Several studies have demonstrated that these compounds can inhibit specific enzymes associated with skin aging [11]. These compounds were also reported to promote the production of essential proteins like collagen by enhancing procollagen synthesis and regulating TGF-β [12,13]. Moreover, they exhibit remarkable anti-inflammatory activity based on their ability to reduce the production of inflammatory mediators via cellular functions, including direct interaction with several receptors, modulation of intracellular signaling, transcription of genes, and modulation of enzymatic activities [14,15,16].

This study investigates the potential of a sugarcane straw extract-based (SSE) ingredient as an effective anti-aging cosmetic ingredient. The research objectives encompass a comprehensive assessment of the ingredient, starting with analyzing its general safety through evaluations of cytotoxicity and sensitization properties. Following that, the efficacy of the SSE ingredient in restraining enzymes associated with skin aging, boosting the production of elastin and hyaluronic acid, and demonstrating anti-pollution properties was evaluated. The SSE ingredient’s ability to permeate the skin was also assessed with an artificial skin membrane model.

## 2. Results and Discussion

### 2.1. Ingredient Characterization

Firstly, ingredient characterization was performed to identify the phenolic compounds responsible for the anti-aging properties. To this end, the sugarcane straw extract on which the ingredient was based was analyzed with LC-ESI-QqTOF-HRMS, and the results are summarized in Table 1. Furthermore, the complete characterization of the extract is described in Carvalho et al. [8].

The hydroxycinnamic acids, with high amounts of *p*-coumaric acid, neochlorogenic acid, and chlorogenic acid, are sugarcane straw extracts most representative phenolic compounds. The second most represented class was hydroxybenzoic acids, with the most common compounds being 1-*O*-vanilloyl-β-D-glucose, 2,4-dihydrobenzoic acid, and 3,4-dihydroxybenzaldehyde. Flavones were the least abundant class, with vitexin and isoorientin being the most abundant compounds within this class.

Some phenolic compounds in sugarcane straw extracts had previously been found in sugarcane juice and molasses extracts. Apigenin-6-C-arabinosyl-8-C-glucoside, tricin 7-*O*-neohesperidoside, tricin 7-*O*-rhamnosyl-glucuronide, and tricin-7-*O*-glycoside were found in an ethanolic molasses extract [17]. Coumaric acid and related flavones such as tricin-7-*O*-rhamnosylglucuronide and apigenin-6-C-glucoside-8-Carabinoside were found in fresh sugarcane juices [18]. Nevertheless, several factors, including genetic variations between cultivars [19] and biotic stressors, including fungal, bacterial, and viral infections and insect attacks, might impact the composition of phenolic compounds in sugarcane straw [20].

The classes of phenolic compounds that are more representative in the extracts are known for their interesting bioactive properties, which could impact the anti-aging capabilities of the ingredient. For instance, protocatechuic acid is reported to possess antioxidant and anti-inflammatory activity [21,22,23], *p*-coumaric acid is known to have antioxidant and anti-inflammatory activity and be capable of inhibiting tyrosinase [16,23,24,25], and chlorogenic acid is known to display antioxidant and anti-inflammatory activity and to inhibit elastase and tyrosinase [21,24,26]. These examples demonstrate the potential anti-aging capacity of this extract.

Identification of compounds was carried out through precise mass and isotope calculations (mSigma, Bruker Daltonics).

### 2.2. Ingredient Safety

Ensuring the safety of cosmetic ingredients for topical application is of paramount importance. Therefore, rigorous evaluations were conducted before assessing the SSE ingredient’s anti-aging properties to determine its cytotoxicity and sensitization potential. These assessments aimed to identify the safe concentration levels that could be used for subsequent analyses.

**Figure 1:**
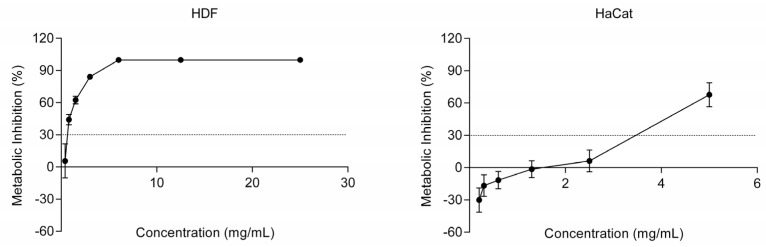
Metabolic activity inhibition assessed with Presto Blue assay of fibroblasts (HDF) and keratinocytes (HaCaT) cells in the presence of increasing concentrations of the SSE ingredient. Cell metabolic activity inhibition results are presented as percentages, where 30% inhibition corresponds to the threshold at which a compound is considered cytotoxic about the negative control (baseline).

Cytotoxicity assessment was performed in two different cell lines: human dermal fibroblasts (HDF), cells found in the dermis, and human keratinocytes (HaCaT), cells found in the epidermis, the outermost layer of the skin [27]. According to ISO 10993-5, concentrations that exhibited a metabolic inhibition below 30% were determined to be non-cytotoxic and, therefore, can be considered safe for further analysis and potential use in cosmetics [28]. As seen in Figure 1, the biocompatible concentration in HDF was determined to be 0.4 mg/mL, while in HaCaT was 2.5 mg/mL. Thus, keratinocytes tolerate a higher concentration of the SSE ingredient when compared to fibroblasts. These differences in cytotoxicity concentrations observed between the HDF and HaCaT cell lines have been previously reported [29].

As previously mentioned, this ingredient was formulated with a sugarcane extract rich in phenolic compounds, namely hydroxybenzoic, hydroxycinnamic, and flavones [8]. Phenolic compounds, specifically hydroxybenzoic acid and vanillic acid, have been reported to not affect the growth of skin fibroblasts until a concentration of 0.2 mg/mL [30]. In a different study, phenolic compounds at 0.01 and 0.03 mg/mL, including phenolic acids, flavanols, and procyanidins, have been reported to inhibit 50% of fibroblasts [29]. This prior research aligns with the findings obtained in our work, where concentrations below 0.4 mg/mL were considered non-cytotoxic in fibroblasts. Regarding keratinocytes, phenolic compounds extracts exhibited a 50% metabolic inhibition in keratinocytes at concentrations of 0.05 and 0.07 mg/mL [29]. Furthermore, it has been shown that a flavonoid-rich shrub extract induces a 50% metabolic inhibition in keratinocytes at a concentration of 0.5 mg/mL [31]. The results indicate that the cytotoxicity concentration obtained for the SSE ingredient matches those found in the literature.

These findings conclude the concentrations that the SSE ingredient is safe to be used in cosmetic formulations.

Sensitization evaluations were performed to assess the SSE ingredient’s potential to induce allergic reactions or sensitization in individuals, ensuring that it would not cause sensitization issues when applied topically. The activation of the adverse outcome pathway, used to measure skin sensitization, occurs in four different events. The Direct Peptide Reactivity Assay (DPRA) is a non-animal, high-throughput screening tool for skin sensitization potential. This assay examines the reactivity of a test compound to two model peptides (cysteine and lysine) and screens for skin sensitization potential [32]. This test method represents the molecular initiating event of the adverse outcome pathway for skin sensitization, in which covalent bonding to skin proteins occurs [33,34]. The results of the DPRA revealed that the SSE ingredient, at a concentration of 1.60 mg/mL, is considered non-sensitizing. However, at higher concentrations, it displays low to moderate reactivity (Table 2). This finding is significant for cosmetic ingredients, as the development of skin sensitization, such as allergic contact dermatitis, has been associated with certain cosmetics [35]. However, these results should be carefully evaluated since plant polyphenols have been observed to generate false positive results in this type of in vitro assay since phenolic compounds can form complexes with peptides, leading to their precipitation [36,37,38]. Thus, other sensitization tests representing different stages of the adverse outcome pathway should be performed.

### 2.3. Skin Aging-Related Enzymes Inhibitory Capacity

**Figure 2:**
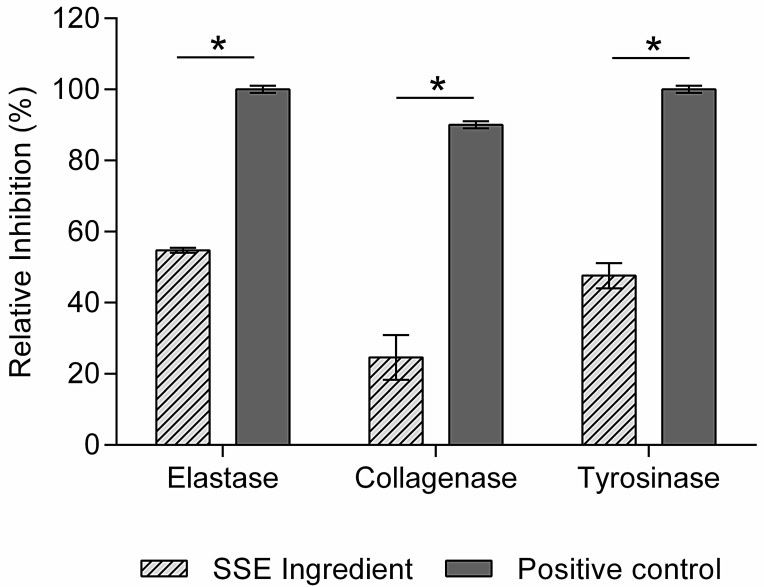
Relative inhibition (%) of elastase, tyrosinase, and collagenase (MMP-1) (*n* = 3) at 2 mg/mL. Elastase positive control was SPCK (5.0 × 10^−4^ mg/mL), collagenase positive control was NNGH (4.1 × 10^−5^ mg/mL), and tyrosinase positive control was Kojic acid (0.021 mg/mL). Asterisks (*) indicate statistical differences (*p* < 0.05).

Phenolic compounds have been found to exhibit inhibitory effects on various skin aging-related enzymes. These enzymes play crucial roles in collagen and elastin degradation and melanin synthesis, contributing to skin aging signs. By inhibiting these enzymes, phenolic compounds can help mitigate the signs of skin aging [39]. The SSE ingredient was screened for inhibiting collagenase, elastase, and tyrosinase. The results showed that the SSE ingredient can inhibit 55% of elastase, 47% of tyrosinase, and 25% of MMP-1 (collagenase) activities (Figure 2) at non-cytotoxic concentrations (2 mg/mL). However, in all cases, the SSE ingredient does not match (*p* < 0.05) the activity of the corresponding positive controls.

Collagenase is an enzyme capable of degrading collagen [11,13]. Collagen is a fundamental protein in the skin’s extracellular matrix structure and the main component of connective tissue, hair, and nails, providing elasticity and strength. During extrinsic aging, ROS initiates a chain reaction of lipid peroxidation in the cell membranes, and due to some signal cascades, an overexpression of matrix MMPs occurs, namely MMP-1 [6]. Elastase is the proteolytic enzyme responsible for the degradation of elastin, causing visible signs of aging like wrinkles, since elastin is the protein responsible for the flexibility of the skin matrix [3]. Phenolic compounds are reported to inhibit the activity of proteinases, which catalyze the degradation of skin proteins, such as collagen and elastin [6]. Besides being responsible for hair and skin color, melanin protects against UV radiation. Tyrosinase is a crucial enzyme for melanin biosynthesis, but it can cause hyperpigmentation due to the accumulation of melanin in specific parts of the skin [3,40]. Due to aromatic purine rings, some phenolic compounds have structures similar to tyrosine, which are oxidized by tyrosinase and can act as substrate analog inhibitors against melanogenesis [41].

Phenolic acids have been reported to display inhibitory activity against these enzymes [16]. Grape pomace from white wine containing phenolic compounds has been reported to inhibit collagenase and elastase; it demonstrated a significant capacity of 50% inhibition for both enzymes at 0.02 mg/mL and 0.01 mg/mL, respectively [42]. A total of nine plant extracts, encompassing white tea, bladderwrack, cleavers, rose tincture, green tea, rose aqueous, angelica, anise, and pomegranate, were identified for their substantial anti-elastase and anti-collagenase potential. These extracts exhibited inhibitory activity ranging from 10% to 89% for elastase and 6% to 87% for collagenase [43]. *Asphodelus microcarpus* leaf, flower, and root extracts rich in phenolic compounds inhibit between 8% and 40% of tyrosinase at 0.2 mg/mL [41].

Based on the information provided, it is reasonable to infer that the SSE ingredient exhibits inhibitory effects on elastase, collagenase, and tyrosinase enzymes at non-cytotoxic concentrations. By inhibiting these enzymes, the SSE ingredient may help preserve skin elasticity and collagen integrity and regulate pigmentation. This suggests that the SSE ingredient has the potential to contribute to anti-aging and skin health benefits.

### 2.4. Impact on Hyaluronic Acid and Elastin Production

Next, we studied the potential of the SSE ingredient to induce elastin and hyaluronic acid production in skin cells.

Loss of skin moisture is a common characteristic associated with skin aging. Hyaluronic acid, a glycosaminoglycan, is crucial role in maintaining skin moisture due to its exceptional ability to attract and retain water molecules [44]. Elastin is a protein responsible for maintaining skin elasticity; thus, when degradation occurs, loss of skin elasticity and the appearance of wrinkles occur [45]. Elastin production was tested in dermal fibroblasts since these cells are responsible for secreting extracellular matrix proteins, including elastin [46]. In contrast, hyaluronic acid was tested in HaCaT since epidermal keratinocytes synthesize this molecule [44].

**Figure 3:**
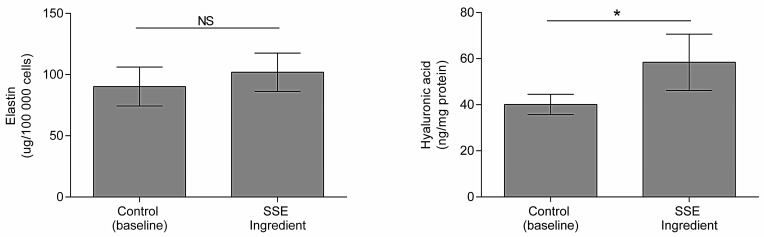
Promotion of elastin and hyaluronic acid (*n* = 3) by HDF (0.3 mg/mL) and HaCaT (1 mg/mL) cells, respectively. Asterisks (*) indicate statistically significant differences (*p* < 0.05). NS indicates that no significant differences (*p* > 0.05) were detected.

Figure 3 demonstrates that the SSE ingredient significantly increases the production of hyaluronic acid (*p* < 0.05) by 46% in HaCaT cells. The observed increase in hyaluronic acid production suggests that the SSE ingredient may improve skin hydration and combat the effects of aging associated with moisture loss.

The SSE ingredient also displays activity towards elastin production, increasing it by 13%, although not significantly (*p* > 0.05) (Figure 3). Phenolic compounds have been reported to increase insoluble elastin deposition [47], although reports on the increase of soluble elastin have not been found.

Therefore, the SSE ingredient, rich in phenolic compounds, might be used in anti-aging cosmetic formulations, as it has been found to promote hyaluronic acid production in the skin.

### 2.5. Cellular Response to Urban Particulate Matter

**Figure 4:**
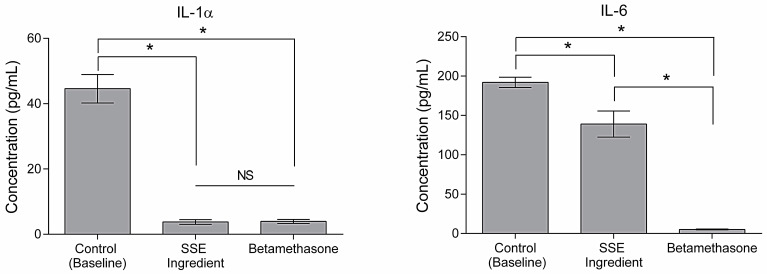
Impact of the SSE ingredient (2.5 mg/mL) in IL-1α and IL-6 concentration (pg/mL) in HaCaT cells exposed to urban particle matter (*n* = 3). Asterisks (*) indicate statistical differences (*p* < 0.05) found between groups. NS indicates that no significant differences (*p* > 0.05) was detected.

Pollution stands out as a primary contributor to extrinsic aging [3]. Consequently, the anti-aging properties of an effective ingredient should encompass robust anti-pollution capabilities. Exposure to pollutants produces ROS and oxidative stress, consequently inducing an inflammatory cascade in the skin and provoking an exacerbated inflammatory response. In this process, the production of pro-inflammatory cytokines such as IL-1α and IL-6 is observed [5]. Thus, one of the strategies to formulate cosmetic ingredients with anti-pollution activity is to develop ingredients with anti-inflammatory potential [48]. As seen in Figure 4, the SSE ingredient decreases IL-1α and IL-6 secretion (*p* < 0.05). Regarding IL-1α, the SSE ingredient matches the activity of betamethasone (positive control). These results are accordingly published where phenolic compounds extracts have been reported to display anti-inflammatory activity by decreasing levels of cytokines [49]. A pomegranate peel extract containing phenolic compounds attenuated the expression of inflammatory cytokines from THP-1 monocytic cells exposed to airborne particulate matter [50]. An aqueous olive extract was reported to decrease the secretion of cytokines, including IL-1α and IL-6, in lipopolysaccharide (LPS)-stimulated macrophages [51]. Furthermore, caffeic acid has been reported to reduce UVB radiation-induced expression of IL-10 [52,53]. Anti-pollution activity has been linked to antioxidant capacity since reducing ROS in the skin can reduce inflammatory response and consequently decrease cytokines, including IL-1α and IL-6 [54]. The sugarcane straw extract that the ingredient is based on displayed antioxidant capacity in a previous study [8]. Thus, this activity may contribute to the SSE ingredient’s anti-pollution activity. Therefore, it is reasonable to deduce that the SSE ingredient manifests anti-pollution properties, augmenting its overall potential for anti-aging benefits.

### 2.6. Permeability

The SSE ingredient’s ability to penetrate the skin was evaluated. Despite its anti-aging properties, an important feature that active ingredients need to have is the capacity to achieve the target skin layer where their biological action could be exerted [6]. Therefore, a permeation assay using a synthetic Strat-M™ membrane was conducted to evaluate this capacity. This membrane allows a non-animal-based model for transdermal diffusion testing that predicts diffusion in human skin without lot-to-lot variability. The SSE ingredient was introduced in the donor chamber of the Franz cells in contact with the membrane and allowed to permeate for 10 h. After this time, the contents of the donor chamber (not absorbed), the receptor chamber, and the Strat-M™ membrane were collected and LC-ESI-QqTOF-HRMS was used to quantify phenolic compounds, and the results are listed in Table 3 and Table 4. A significant proportion of the identified phenolic compounds, including hydroxybenzoic acids, hydroxycinnamic acids, and flavones, were found to be retained in the membrane surface, with percentages ranging from 59 to 67% (Table 3). Only 17 to 25% of the phenolics present in the SSE ingredient were absorbed by the membrane, while 8 to 23% reached the receptor cell, meaning they passed the membrane. Flavones were found to be the most predominant group among the phenolic compounds present in the membrane, accounting for approximately 25%.

Table 4 shows each class’s most representative phenolic compounds and their distribution throughout the permeation system. Among the individual compounds, chlorogenic acid and *p*-coumaric acid, both hydroxycinnamic acids, were identified as the most abundant in the membrane. Luteolin-8-C-glucoside, a flavone, was also notably present in higher quantities within the membrane. These results indicate that while certain phenolic compounds, such as flavones, exhibited a higher presence in the membrane, most of the phenolic compounds tested did not demonstrate significant absorption into the membrane. This suggests that these compounds have limited permeability across the membrane barrier.

Regarding phenolic compounds’ activity as anti-aging molecules, they should be able to reach the epidermis and the dermis, where elastin, collagen fibers, and the hyaluronic acid matrix are located [55]. However, the permeation of cosmetic ingredients through the skin into the vascular system is not desirable [6]. In this experience, the membrane simulates the skin barrier. Therefore, it is possible to extrapolate that most of the phenolic compounds present in the SSE ingredient remain on the skin’s surface, and only a smaller percentage are retained by the skin. Furthermore, an even smaller percentage (8–23%) can permeate through the skin and reach the vascular system, in which their activity is unknown. Some reasons for the lack of capacity of phenolic compounds to cross the skin membrane are poor aqueous solubility, low availability, rapid metabolism, and systemic elimination [56].

Nevertheless, different phenolic compounds have different permeability capacities. Molecular weight, size, and lipophilic characteristics are some factors that influence phenolic compounds permeability [57,58]. The Strat-M™ membrane is the most suitable synthetic membrane to simulate human skin for active compounds or drug diffusion experiments. It comprises two layers of polyethersulfone lying on top of another layer of polyolefin, meaning that the polyethersulfone layer is more resistant to diffusion. In contrast, the polyolefin layer is more diffusive [59]. It also contains a combination of lipids (ceramides, cholesterol, free fatty acids, and other components) in a specific ratio similar to what is found in the human *Stratum corneum* [60]. This lipid structure can affect the permeability of specific compounds. Since hydroxycinnamic and hydroxybenzoic acids are more polar compounds, these cannot easily cross the membrane.

On the other hand, flavones contain lipophilic (non-polar) as well as hydrophilic (polar) fragments [61], and thus these should permeate more quickly through the membrane. Thus, flavones are the class of phenolic compounds that more easily cross the skin membrane, which is supported by previous findings [62]. Conversely, chlorogenic acid has been reported to have low skin permeability [62], which was not observed in this work. The methodology itself can be a factor to consider. A previous study using a skin model with pig ears reported that hydroxycinnamic acids and gentisic acid were highly retained in the skin, and the higher content for those compounds was detected in the receptor medium (16–36%) [63]. However, some strategies can be employed to increase the absorption of the phenolic compounds by the skin, such as employing the SSE ingredient in an emulsion, liposomes, and nanoparticles, since chemical compounds can decrease the barrier function [64].

Understanding the absorption and retention characteristics of phenolic compounds in the membrane is essential for evaluating their potential bioavailability and activity. These findings highlight the specific phenolic compounds that have a higher affinity for the membrane and contribute to our understanding of their behavior about membrane permeability and cellular uptake. Furthermore, to validate these results, ex vivo and in vivo studies would help evaluate the efficacy and safety of these strategies, allowing for a comprehensive assessment of the skin penetration and bioavailability of phenolic compounds.

## 3. Materials and Methods

### 3.1. Material

The development of the SSE ingredient was previously described in [8]. In short, straw provided by Raízen from São Paulo, Brazil, underwent a milling and drying process. A solid–liquid extraction using 50% ethanol (*v*/*v*) (Honeywell, Morris Plains, NJ, USA) was performed, followed by purification using amberlite XAD-2. To obtain a powder, the resulting straw extract was freeze-dried (Martin Christ, Osterode am Harz, Germany). The SSE ingredient was obtained by mixing the dry sugarcane straw extract (20% wt) with 1,2-hexanediol (Sigma-Aldrich, St. Louis, MO, USA), a cosmetic solvent, in a 25:75 ratio (*w*/*v*) with water.

### 3.2. Phenolic Compounds Identification and Quantification Using LC-ESI-QqTOF-HRMS

The SSE ingredient major bioactive compounds were analyzed with Liquid Chromatography—Electrospray Ionization—Ultrahigh-Resolution—Quadrupole Time of Flight—Mass Spectrometry (LC-ESI-UHR-QqTOF-MS) as described by Oliveira et al., (2015) [65].

Briefly, using a Bruker Elute series with a UHR-QqTOF mass spectrometer (Impact II, Bruker Daltonics, Bremen, Germany) and a BRHSC18022100 intensity Solo 2 C18 column (100 × 2.1 mm, 2.2 µm, Bruker), the separation was completed over 26 min at a flow rate of 0.25 mL/min. The parameters for MS assessment were set using negative ionization mode with spectra acquired over a range from *m*/*z* 20 to 1000 in the Auto MS scan mode. The elemental composition was confirmed based on accurate mass and isotope rate calculations designated as mSigma (Bruker Daltonics), and phenolic compounds were identified using their accurate mass [M-H]^−^ using the Bruker Compass DataAnalysis software (version 5.1, Bruker Daltonic GmbH, Billerica, MA, USA). This identification was performed with a previous internal mass calibration with sodium formate clusters.

Quantification was achieved with calibration curves of the standards, apigenin, isoschaftoside, orientin/luteolin-8-C-glucoside, vitexin/apigenin-8-C-glucoside, vitexin-2-*O*-rhamnoside, luteolin (Extrasynthése, France), diosmetin, naringenin, vanillin, 4-caffeoylquinic acid tricin, naringenin-7-*O*-glucoside/prunin, protocatechuic acid, vanillic acid, *p*-coumaric acid, caffeic acid, ferulic acid, chlorogenic acid, gentisic acid, 4-hydroxybenzaldehyde, 4-hydroxybenzoic acid, 3,4-dihydroxybenzalhedyde, citric acid, quinic acid, malic acid, aconitic acid, sebasic acid, and azelaic acid (Sigma-Aldrich, St. Louis, MO, USA). The results are expressed in µg/g dw of extract. The results are expressed in µg/g dw of extract.

### 3.3. Cytotoxicity

Normal human dermal fibroblasts (HDF) from adult skin (Lonza Bioscience, Cat. CC2511) and immortalized human keratinocytes (HaCaT) (CLS) were cultured in Gibco Dulbecco’s Modified Eagle Medium (DMEM) supplemented with 10% fetal bovine serum (FBS) (10082-147, Gibco, New York, NY, USA), and 1% antibiotic-antimycotic (15240-062, Gibco, New York, NY, USA). Cells were maintained at 37 °C in a 5% CO_2_ humidified atmosphere. Cells were harvested using TrypLE™ Express Enzyme (1X). Cells were resuspended and counted using 0.4% Trypan Blue (T8154-20ML, Sigma-Aldrich, St. Louis, MO, USA) and the Countess II FL Automated Cell Counter (Invitrogen, Waltham, MA, USA). Cell viability in response to the SSE ingredient was assessed using the Presto Blue cell viability reagent (A13262, Invitrogen, MA, USA). For that, cells were seeded at 1 × 10^5^ cells/mL in a 96-well plate. After 24 h, cells were exposed to the SSE ingredient and incubated for 24 h. A 10% dimethyl sulfoxide (DMSO) solution in a culture medium was used to control complete metabolic inhibition. Then, Presto Blue cell viability reagent was added to each well at a 1:10 ratio, and cells were incubated for 1 h. Fluorescence was measured in a Synergy H1 microplate reader (Biotek, Winooski, VT, USA) at an excitation of 560 nm and an emission of 590 nm. Experiments were performed in triplicate, expressing the results as a percentage of cell metabolic inhibition. A cytotoxic effect is assumed for a metabolic inhibition superior to 30%, as described by ISO 10993-5 [28].

### 3.4. Direct Peptide Reactivity Assay

Direct peptide reactivity assay (DPRA) was performed according to the OECD 442C guidelines [66]. Briefly, dissolved test substances were incubated with peptides in ratios of 1:10 (for cysteine peptide) or 1:50 (for lysine peptide) for 24 h at room temperature, and the remaining non-depleted peptide concentration was determined using high-performance liquid chromatography (HPLC) with gradient elution and UV-detection at 220 nm. Test substances previously dissolved in an appropriate solvent were incubated in ratios of 1:5 for cysteine peptide or 1:24 for lysine peptide. Further, a co-elution control was performed with each test substance to detect possible interference with the peptides. Cysteine and lysine peptide depletion relative to solvent control were reported for each test substance. In contrast, negative values from 0 to −10% were considered no depletion, i.e., reported as 0%, and negative values below −10% were reported as unmodified as they indicate co-elution. Cysteine and lysine percent peptide depletion values were used to categorize a substance in one of four reactivity classes to support the discrimination between skin sensitizers and non-sensitizers (Table 5).

### 3.5. Evaluation of the Inhibition of Skin Aging-Related Enzymes

#### 3.5.1. Collagenase

The screening for collagenase inhibitors was performed using a metalloproteinases-1 (MMP-1) Inhibitor Screening Assay Kit (colorimetric) (1b139443) (Abcam, Cambridge, UK). MMP inhibitor, MPP substrate, and MPP1 enzyme were prepared according to instructions. The MMP-1 enzyme was introduced to a flat-bottom microplate (Thermo Scientific, Waltham, MA, USA), and then various samples, including blank (assay buffer), control, MMP inhibitor, and test samples, were added. The plate was incubated at 37 °C for 1 h to facilitate the interaction between the inhibitor and enzyme. Subsequently, the reaction was initiated by adding the MMP-1 substrate, and the optical density (OD) was measured at 412 nm using a Synergy H1 microplate reader at intervals of 10 to 20 min. N-Isobutyl-N-(4-methoxyphenylsulfonyl) glycyl hydroxamic acid (NNGH) (4.1 × 10^−5^ mg/mL) was tested as a positive control. A range of time points, during which the reaction was linear, was selected to obtain the reaction velocity (v), and then the inhibitor activity was calculated through the following formula:

(1) $$\mathrm{Inhibitor}\%\;\mathrm{activity}\;\mathrm{remaining}=\frac{V\;\mathrm{inhibitor}\;}{V\;\mathrm{control}}\times 100$$

#### 3.5.2. Elastase

Elastase inhibitor screening was performed with a Neutrophil Elastase Inhibitor Screening Kit (fluorometric) (ab118971) (abcam, Cambridge, UK). Initially, the neutrophil elastase (NE) enzyme was prepared according to instructions and added to a microplate with a flat bottom. Then, test inhibitor, inhibitor control, and blank were added, and the plate was incubated for 5 min at 37 °C. After this period, the substrate reaction mix was prepared and added. The plate was incubated in a microplate reader at 37 °C for 30 min, and the fluorescence was measured at 400 and 505 nm. The peptide succinyl-alanyl-alanyl-prolyl-valine chloromethyl ketone (SPCK) was tested as a positive control at 5.0 × 10^−4^ mg/mL. Afterward, two-time points in which the reaction was linear were selected to calculate the relative activity for each test inhibitor according to the following formula, in which RFU is the fluorescence generated by the hydrolyzation of the substrate:

(2) $$\%\;\mathrm{relative}\;\mathrm{activity}=\frac{\Delta \mathrm{RFU}\;\mathrm{test}\;\mathrm{inhibitor}\;}{\Delta \mathrm{RFU}\;\mathrm{enzyme}\;\mathrm{control}}\times 100$$

#### 3.5.3. Tyrosinase

The screening for tyrosinase inhibitors was performed with a Tyrosinase Inhibitor Screening kit (colorimetric) (ab204715) (abcam, Cambridge, UK). Firstly, 20 µL of sample solutions were placed in a microplate with a flat bottom along with a tyrosinase inhibitor working solution (Kojic acid), and a tyrosinase assay buffer. The tyrosinase enzyme solution was prepared, and 50 µL was added to test solutions. The plate was incubated at 25 °C for 10 min. Afterward, 30 µL of tyrosinase substrate solution was added, the plate was incubated at 25 °C for 1 h, and the OD was measured in a microplate reader every 2 to 3 min at 510 nm. Kojic acid (0.021 mg/mL) was tested as a positive control. After the readings, two time points were selected to calculate the slope for all samples, inhibition control (IC), and enzyme control (EC), and the relative inhibition was calculated through the following formula:

(3) $$\%\;\mathrm{Relative}\;\mathrm{Inhibition}\;=\frac{\mathrm{Slope}\;\mathrm{EC}-\mathrm{Slope}\;\mathrm{sample}\;}{\mathrm{Slope}\;\mathrm{EC}}\times 100$$

### 3.6. Hyaluronic Acid Quantification

HaCaT cells were seeded in 12-well plates at 1.5 × 10^5^ cells/mL for 24 h. The cells were then exposed to the SSE ingredient (1 mg/mL) and incubated for 24 h. Cell culture supernatants were collected to quantify hyaluronic acid using the Quantikine™ ELISA Hyaluronan Immunoassay (R&D Systems Inc., Minneapolis, MN, USA) according to the manufacturer’s instructions. Additionally, cells were harvested and lysed for protein extraction using lysis buffer (50 mM Tris-HCl, pH 7.8, 150 mM NaCl, 1% Nonidet-P40, and protease inhibitor cocktail tablets (Roche, Basel, Switzerland)). Total protein content was quantified using a Pierce™ BCA Protein Assay Kit (Thermo Fisher, MA, USA). All assays were performed in triplicate. The results are expressed as ngHA/mgprotein.

### 3.7. Elastin Quantification

HDF cells were seeded in 6-well plates at 1.5 × 10^5^ cells/mL for 24 h. Cells were then exposed to the SSE ingredient (0.3 mg/mL) and incubated for 48 h, after which the medium was replaced with fresh medium containing the same concentration of the active ingredient for an additional 24 h. After that, cells were harvested and counted with 0.4% Trypan Blue using a Countess II FL Automated Cell Counter. According to the manufacturer’s instructions, soluble elastin was extracted from cells with 0.25 M oxalic acid and quantified using the Fastin™ Elastin assay kit (Biocolor, Co Antrim, UK). All assays were performed in triplicate. The results are expressed as µg Elastin/1 × 10^5^ cells.

### 3.8. Exposure to Urban Particle Matter

HaCaT cells were seeded at 1 × 10^5^ cells/well in a 24-well plate and incubated for 24 h. Then, cells were exposed to 500 µg/mL of urban particle matter (SRM1648a NISTS) plus the SSE ingredient (2.5 mg/mL) for 24 h. Afterward, medium supernatants were collected and used to evaluate the levels of secreted proinflammatory cytokines IL1-α and IL-6 using an ELISA MAX™ Deluxe Set Human IL-1α and ELISA MAX™ Deluxe Set Human IL-6 (Biolegend, San Diego, CA, USA), respectively. The results are expressed in pg of cytokine/mL. Two independent experiments were performed.

### 3.9. In Vitro Penetration with a Synthetic Membrane

An in vitro study was conducted to study the penetration of phenolic compounds, according to the OECD 428 guidelines [67]. A polyethersulfone synthetic membrane (Strat-M™) (Merck-Millipore, Burlington, MA, USA) was used. This synthetic membrane is composed of three layers: the top layer resembles the stratum corneum, then two layers of polyethersulfone that resemble the dermis, both of which are resistant to diffusion, and finally, one layer of polyolefin that resembles the subcutaneous fat tissue, which is more open and diffusive.

The permeation assay was carried out on Franz diffusion cells with a skin exposure area of 0.9 cm^2^, 1.5 cm headspace height, and receptor volume of 5 mL. Two jacketed cells were mounted in a magnetic stirrer and kept at 32 °C to mimic human skin surface temperature using circulating water from a thermostatic water bath. The receptor solution was composed of phosphate-buffered saline (PBS) (pH 7.4) and was equipped with small, Teflon-coated magnets, keeping the receptor medium thoroughly stirred during the entire experiment.

The SSE ingredient (100 μL) was directly applied to the membrane, and the permeation process was conducted for 10 h. At the end of the experiment, the sample present on the membrane surface was washed with water, and both the washed sample and the membrane were collected for further analysis. The synthetic membrane was divided into pieces, and the retained phenolic compounds were extracted using 50% ethanol with agitation overnight. Subsequently, the ethanol evaporated. The remaining PBS solution in the receptor chamber was also collected. The three fractions were concentrated using a freeze-drier and then resuspended in water to identify and quantify phenolic compounds via LC-ESI-QqTOF-HRMS, as described in Section 3.2.

### 3.10. Statistical Analysis

Statistical analysis was performed using IBM SPSS statistics (v. 21, 2012) (New York, NY, USA) software. The Shapiro–Wilk test was performed to determine whether the data displayed a normal distribution. The *t*-student and one-way ANOVA tests were used when a normal distribution was detected, and a 5% confidence level was used to determine whether the differences were statistically significant.

## 4. Conclusions

The cosmetics industry has been focusing on developing sustainable and natural-origin ingredients that have less adverse effects on the skin. The SSE ingredient tested in vitro in this work has shown anti-aging properties, namely inhibition of collagenase, elastase, and tyrosinase and promoting of hyaluronic acid production at non-cytotoxic and low sensitizer concentrations. The SSE ingredient also displays anti-pollution activity by significantly decreasing IL1-α and IL-6 cytokines in HaCaT cells exposed to pollution particles. This indicates that the SSE ingredient can potentially mitigate the inflammatory response induced by pollution exposure. Based on the findings of the permeabilization study using a synthetic membrane, it has been observed that the phenolic compounds present in the SSE ingredient primarily reach the skin’s surface. These results are not entirely desirable since the target of these compounds is not only the epidermis but also the dermis; thus, strategies to increase permeation should be investigated.

## Figures and Tables

**Table 1:** LC-ESI-UHR-QqTOF-MS data of phenolic compounds identified in ethanolic extracts obtained from sugarcane straw and concentration (µg/g dw extract, mean ± SD) of the compound.

Tentative Identification	Formula-H	Retention Time (min)	*m*/*z* Measured [M-H]	MS/MS Fragments (*m*/*z*)	Concentration (µg/g dw Extract)
Hydroxybenzoic acids					
1-*O*-Vanilloyl-β-D-glucose	C_14_H_17_O_9_	6.4	329.0	167	109.7 ± 5.7
Vanillic acid	C_8_H_7_O_4_	7.5	167.0	108, 119, 152	28.03 ± 6.27
Protocatechuic acid	C_7_H_5_O_4_	5.5	153.0	109, 153	14.01 ± 0.69
2,5-Dihydrobenzoic acid	C_7_H_5_O_4_	7.8	153.0	109, 153	10.31 ± 0.75
2,4-Dihydrobenzoic acid	C_7_H_5_O_4_	8.9	153.0	109, 153	441.7 ± 38.3
Gentisic acid 2-*O*-β-glucoside	C_13_H_15_O_9_	5.4	315.1	108, 152	21.51 ± 4.71
Gentisic acid 5-*O*-β-glucoside	C_13_H_15_O_9_	5.9	315.1	109, 153	23.11 ± 3.28
4-Hydroxybenzoic acid	C_7_H_5_O_3_	6.9	137.0	137	12.44 ± 0.38
3,4-Dihydroxybenzaldehyde	C_7_H_5_O_3_	7.3	137.0	93, 137	28.20 ± 0.95
4-Hydroxybenzaldehyde	C_7_H_5_O_2_	8.7	121.0	121	33.66 ± 1.39
Hydroxycinnamic acids					
Neochlorogenic acid	C_16_H_17_O_9_	6.3	353.1	135, 179, 191	250.9 ± 18.5
Chlorogenic acid	C_16_H_17_O_9_	7.8	353.1	191	262.4 ± 12.1
4-Caffeoylquinic acid isomer 1	C_16_H_17_O_9_	8	353.1	135, 173, 179, 191	122.9 ± 5.4
4-Caffeoylquinic acid isomer 2	C_16_H_17_O_9_	9.1	353.1	191	25.63 ± 2.25
3-*O*-Feruloylquinic acid	C_17_H_19_O_9_	8	367.1	134, 193	208.6 ± 6.6
*trans*-3-Feruloylquinic acid	C_17_H_19_O_9_	9.7	367.1	173	100.9 ± 2.0
Caffeic acid	C_9_H_8_O_4_	8.2	179.0	135, 179	18.82 ± 0.62
Isoferulic acid	C_10_H_9_O_4_	11.1	193.0	134, 161, 193	21.01 ± 0.48
*p*-Coumaric acid	C_9_H_7_O_3_	10.3	163.0	119	145.4 ± 5.7
1,3-Dicaffeoylquinic acid	C_25_H_23_O_12_	12.1	515.1	173, 179, 191, 335, 353	13.61 ± 0.59
Flavones					
Apigenin-8-C-glucoside isomer 1	C_21_H_19_O_10_	8.6	431.1	89, 179	2.773 ± 0.269
Apigenin-8-C-glucoside isomer 2	C_21_H_19_O_10_	9	431.1	311, 341, 431	10.46 ± 0.48
Apigenin-6-C-glucoside	C_21_H_19_O_10_	11	431.1	311, 341	6.094 ± 0.352
Isoschaftoside	C_26_H_27_O_14_	10.1	563.1	353, 473	5.643 ± 0.334
Luteolin-8-C-glucoside	C_21_H_19_O_11_	10.3	447.1	327, 357	16.45 ± 0.62
Vitexin 2″-*O*-beta-L-rhamnoside	C_27_H_29_O_14_	11.2	577.2	293, 413	4.153 ± 0.159
Apigenin 7-*O*-neohesperidoside isomer 2	C_27_H_29_O_14_	13.9	577.2	293, 413, 474	6.410 ± 0.180
Tricin-*O*-neohesperoside isomer 2	C_29_H_33_O_16_	13.3	637.2	329	2.748 ± 0.137
Tricin-7-*O*-glucoside	C_25_H_31_O_10_	12.4	491.2	329	6.420 ± 0.095
Tricin	C_17_H_13_O_7_	17.7	329.1	299	7.949 ± 0.236

**Table 2:** Results for sensitization according to the DPRA assay for the SSE ingredient (*n* = 3).

Sample	mg/mL	% Cysteine and Lysine Peptides Depletion	Reactivity (Cysteine and Lysine)	Based on the Mean of Cysteine and Lysine Prediction Model	Potential Sensitizer
Positive Control (Cinnamic aldehyde)	0.1 M	78.8	High reactivity	Positive	Yes
SSE ingredient	12.5	29.3	Moderate	Positive	Yes
	6.25	16.0	Low	Positive	Yes
	3.15	8.7	Low	Positive	Yes
	1.60	4.3	Minimal	Negative	No

**Table 3:** Phenolic compounds quantity (%) that were not absorbed and retained in the Strat-M™ membrane and that passed the membrane into the receptor cell after 10 h (*n* = 3).

	Not Absorbed (%)	Strat-M™ (%)	Receptor Cell (%)
Hydroxybenzoic acids	59.03	17.06	23.91
Hydroxycinnamic acids	67.10	18.70	17.60
Flavones	66.70	25.10	8.20

**Table 4:** Phenolic compounds average concentration (mean ± SD) (µg) applied to the Franz cell, the unabsorbed part, the compounds retained in the Strat-M™ membrane, and the compounds that pass through the membrane into the receptor cell. Different letters indicate statistical differences (*p* < 0.05) (*n* = 3).

	Applied Dose (µg)	Not Absorbed (µg)	Strat-M™ (µg)	Receptor Cell (µg)
Hydroxybenzoic acids	24.27 ± 3.89 ^a^	14.60 ± 1.97 ^b^	4.45 ± 1.80 ^c^	7.56 ± 1.53 ^c^
1-*O*-Vanilloyl-β-D-glucose	6.13 ± 1.18 ^a^	6.04 ± 0.23 ^a^	1.29 ± 0.69 ^b^	0.78 ± 0.26 ^b^
2,5-Dihydroxybenzoic acid	6.04 ± 0.99 ^a^	3.39 ± 0.45 ^b^	1.15 ± 0.27 ^c^	1.35 ± 0.64 ^c^
4-Hydroxybenzaldehyde	6.32 ± 0.80 ^a^	1.59 ± 0.85 ^b^	1.71 ± 0.61 ^b^	2.41 ± 1.44 ^b^
Hydroxycinnamic acids	48.04 ± 8.09 ^a^	41.00 ± 1.29 ^a^	8.22 ± 2.92 ^b^	10.32 ± 1.65 ^b^
Chlorogenic acid	8.15 ± 1.57 ^a^	7.40 ± 0.16 ^a^	1.86 ± 0.99 ^b^	0.78 ± 0.13 ^b^
Ferulic acid	1.21 ± 0.11 ^a^	0.65 ± 0.14 ^b^	0.49 ± 0.16 ^b^	0.66 ± 0.35 ^ab^
*p*-Coumaric acid	4.43 ± 0.63 ^a^	1.90 ± 0.78 ^b^	1.74 ± 0.59 ^b^	2.86 ± 1.38 ^ab^
Flavones	44.51 ± 2.65 ^a^	43.09 ± 0.37 ^a^	14.25 ± 2.07 ^b^	6.64 ± 1.16 ^c^
Apigenin-8-C-glucoside	5.76 ± 0.81 ^a^	5.34 ± 0.11 ^a^	0.92 ± 0.58 ^b^	0.78 ± 0.15 ^b^
Isoschaftoside	5.16 ± 0.60 ^a^	5.05 ± 0.10 ^a^	1.40 ± 0.86 ^b^	0.67 ± 0.13 ^b^
Luteolin-8-C-glucoside	3.13 ± 0.17 ^a^	3.31 ± 0.03 ^a^	1.85 ± 0.65 ^b^	0.63 ± 0.20 ^c^

**Table 5:** DPRA prediction model.

Mean of Cysteine and Lysine % Depletion	Reactivity Class	DPRA Prediction
0% ≤ Mean % Depletion ≤ 6.38%	No or Minimal Reactivity	Negative
6.38% < Mean % Depletion ≤ 22.62%	Low Reactivity	Positive
22.62% < Mean % Depletion ≤ 42.47%	Moderate Reactivity	
42.47% < Mean % Depletion ≤ 100%	High Reactivity	

## Data Availability

Data will be made available upon request.
